# Supplementary material for: Construction of a Recombinant Porcine Epidemic Diarrhea Virus Encoding Nanoluciferase for High-Throughput Screening of Natural Antiviral Products
Source: Viruses. 2021 Sep 18;13(9):1866. doi: 10.3390/v13091866 (PMC8473292; doi:10.3390/v13091866)
Supplement: Supplementary file 1 [file viruses-13-01866-s001.zip › Figure S1.pdf]

# Figure S1

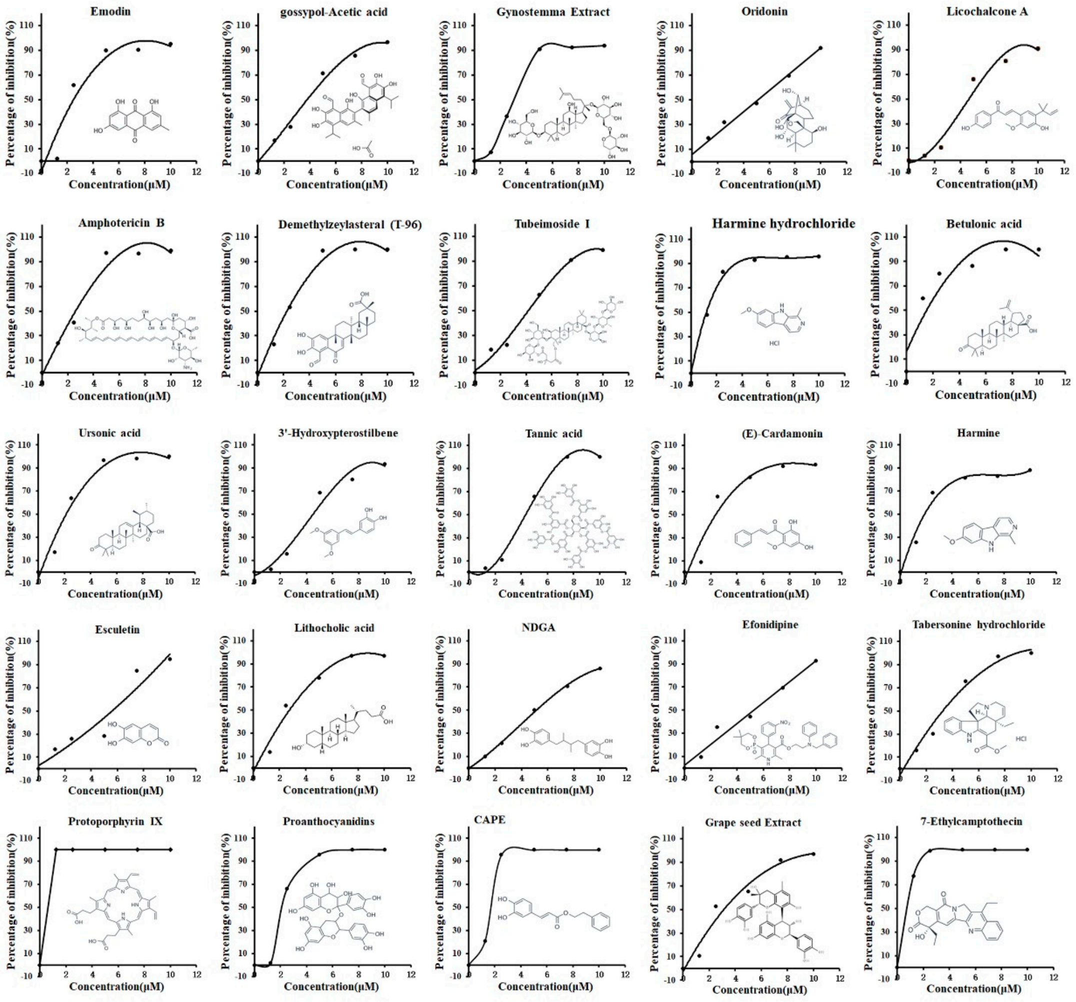


**Fig S1.** Dose-response curves for 25 inhibitors of PEDV in vitro. Vero cells were treated with five doses (1.25μM,2.5μM,5μM,7.5μM and 10μM) of 25 hit compounds for 1h respectively, and then infected with rPEDV-NLuc at a MOI of 0.01. At 20 h post-infection, the cell lysates were assessed for luminescent signals.
