# Supplementary material for: Construction of a Recombinant Porcine Epidemic Diarrhea Virus Encoding Nanoluciferase for High-Throughput Screening of Natural Antiviral Products
Source: Viruses. 2021 Sep 18;13(9):1866. doi: 10.3390/v13091866 (PMC8473292; doi:10.3390/v13091866)
Supplement: Supplementary file 1 [file viruses-13-01866-s001.zip › Table S1.pdf]

Table S1. Primers used for viral genome sequencing in this study.

Primer	Location[a]	Sequence（5'-3'）
1F	1-24	ACTTAAAGAGATTTTCTATCTATG
1R	1232-1255	CACCTTAAGAATTCGCCAGAATGC
2F	1062-1083	ATTGGACTTCCTATGTCTCCAC
2R	2124-2145	ACACAAAGCTCTGCAACACTAG
3F	1986-2008	TCTTCGAGAGTGACGGGTTTTAC
3R	3137-3161	CCTCAATGGCAACGTTGATATACTC
4F	2973-2996	GTGGTGGTGATGTCAAATTCTCTG
4R	4198-4220	CAAGGTCACTAAAATCTCCTTGG
5F	4049-4072	GGTTATGGTCGTCATCAGATAAAG
5R	5229-5253	GTAACAGTGGATTCTACTTTAACAG
6F	5053-5075	CAAGGTATCTAAGCTTAAAGGCA
6R	6251-6273	GGTACACCAGCTAGAACTTTAAC
7F	6059-6082	CAGACGGCTGTTGTGATTAAAGAC
7R	7266-7290	GCATTCTTAACCTGATTTACATTGG
8F	7089-7111	GTCCTGCCACTATTCTTATTGAC
8R	8255-8277	CAAGCCGAATTAAAAATGCAAGC
9F	8103-8125	CTATAGTCGTTGGTGTATCAGAC
9R	9268-9290	AGCCAGCCTGTAGAGTTGAATTG
10F	9107-9128	CTGCGTCAGTATGCTAGTACTT
10R	10275-10298	CAGGATTTACCCAAAAGAACTTAG
11F	10120-10141	CTATACCTCGTTGACAGATGAG
11R	11314-11336	GGCGTGCATTTTCATAAATGACA
12F	11151-11172	TGTGTAATGACCCAGAAAAAGC
12R	12319-12341	CATTACCAGAACCATTGGCCAAC
13F	12162-12182	ACATAGGTGCCACTGTACGCT
13R	13246-13268	GGTATACCCATTCCCTTGATGCT
14F	13037-13060	CCTACTTCAATAATAAAGTCTGGT
14R	14273-14294	TGATGATACTGCCGAGTAGTCA
15F	14088-14110	TCCTTTGAACAAGTTTGGTAAAG
15R	15300-15323	CAGAATTTAGCAGTAGAATCTTCC
16F	15141-15163	GCTTGAACGTTATGTGTCATTGG
16R	16308-16328	CAAAGTGAATCGACCGCTGCA
17F	16146-16167	CATACCTGAAGCTTATTCTAGC
17R	17307-17331	CGAGATAACATGCTCATATTTAATG
18F	17142-17164	GCTTAAATTGTCTGATTTGCAGG
18R	18287-18309	CACATTACAATTCCAAAACAAGC
19F	18121-18143	CTTAAGTTATACAATCCGAAAGC
19R	19294-19316	ATGTCGCTACGAGGGCTAAAATC
20F	19144-19166	ACTGGCATAAAGTTAACTTATGG
20R	20290-20312	GTACAGAACATTGTCCAATACTC

[a] Location corresponds to position within the genome of PEDV YN150.

Continued Table S1

Primer	Location[a]	Sequence（5’-3’）
21F	20078-20101	CCTTATACCTGTCAGATAAGTTTG
21R	21232-21255	GTTCGTAAACATATTGCATAGCAC
22F	21072-21094	CTATTTAACAAAGCCATCCCAGC
22R	22260-22284	GTGAAATGGTAAATTGTCTAGTGTC
23F	22034-22054	CCAACTCAAGTGTTCTCAGGT
23R	23249-23269	GAAACACCCAGCACATTAGTA
24F	23103-23123	TACACTGCAGCATGTAAGACC
24R	24255-24275	AACGGTAGGTTTTCTAGGTTC
25F	24044-24064	CATTTTCTCTCTGGTACAGGC
25R	25287-25308	CCCACGTATAGCTAGATACAAG
26F	25124-25144	TGGCGCTATAAAAATGCGCTC
26R	26288-26310	GATTACTCACAGCTGAGTAGTCG
27F	26104-26124	CACTCCTTAGTGGTACATTGC
27R	27266-27288	GCATCTCCAAAATTTTTGAAGCC
28F	27053-27074	GCCCTTAAATCTTTGGGTATTG
28R	28006-28028	GTGTATCCATATCAACACCGTCA

[a] Location corresponds to position within the genome of PEDV YN150.
